# *Mesodinium–Dinophysis* encounters: temporal and spatial constraints on *Dinophysis* blooms

**DOI:** 10.1093/plankt/fbae068

**Published:** 2025-03-16

**Authors:** Patricio A Díaz, Ángela M Baldrich, Francisco Rodríguez, Manuel Díaz, Gonzalo Álvarez, Iván Pérez-Santos, Camila Schwerter, Camilo Rodríguez-Villegas, Pamela Carbonell, Bárbara Cantarero, Loreto López, Beatriz Reguera

**Affiliations:** Centro i~mar, Universidad de Los Lagos, Camino Chinquihue Km 6, Casilla 557, 5480000 Puerto Montt, Chile; CeBiB, Universidad de Los Lagos, Camino Chinquihue Km 6, Casilla 557, 5480000 Puerto Montt, Chile; Centro i~mar, Universidad de Los Lagos, Camino Chinquihue Km 6, Casilla 557, 5480000 Puerto Montt, Chile; CeBiB, Universidad de Los Lagos, Camino Chinquihue Km 6, Casilla 557, 5480000 Puerto Montt, Chile; Centro Oceanográfico de Vigo, Instituto Español de Oceanografía (IEO-CSIC), Subida a Radio Faro 50, 36390 Vigo, Spain; Programa de Investigación Pesquera, Instituto de Acuicultura, Universidad Austral de Chile, Los Pinos s/n, 5480000 Puerto Montt, Chile; Facultad de Ciencias del Mar, Departamento de Acuicultura, Universidad Católica del Norte, Larrondo 1281, 1780000 Coquimbo, Chile; Centro de Investigación y Desarrollo Tecnológico en Algas (CIDTA), Facultad de Ciencias del Mar, Universidad Católica del Norte, Larrondo 1281, 1780000 Coquimbo, Chile; Center for Ecology and Sustainable Management of Oceanic Islands (ESMOI), Facultad de Ciencias del Mar, Universidad Católica del Norte, Larrondo 1281, 1780000 Coquimbo, Chile; Centro i~mar, Universidad de Los Lagos, Camino Chinquihue Km 6, Casilla 557, 5480000 Puerto Montt, Chile; Centro de Investigación Oceanográfica COPAS COASTAL, Universidad de Concepción, Barrio Universitario s/n, 4030000 Concepción, Chile; Centro de Investigaciones en Ecosistemas de la Patagonia (CIEP), Camino Coyhaique Alto Km 4, 5950000 Coyhaique, Chile; Centro i~mar, Universidad de Los Lagos, Camino Chinquihue Km 6, Casilla 557, 5480000 Puerto Montt, Chile; Centro i~mar, Universidad de Los Lagos, Camino Chinquihue Km 6, Casilla 557, 5480000 Puerto Montt, Chile; Centro de Estudios de Algas Nocivas (CREAN), Instituto de Fomento Pesquero (IFOP), Padre Harter 574, 5480000, Puerto Montt, Chile; Centro i~mar, Universidad de Los Lagos, Camino Chinquihue Km 6, Casilla 557, 5480000 Puerto Montt, Chile; Centro de Estudios de Algas Nocivas (CREAN), Instituto de Fomento Pesquero (IFOP), Padre Harter 574, 5480000, Puerto Montt, Chile; Centro Oceanográfico de Vigo, Instituto Español de Oceanografía (IEO-CSIC), Subida a Radio Faro 50, 36390 Vigo, Spain

**Keywords:** *Dinophysis acuminata* complex, *Mesodinium rubrum/M. major* complex, *Dinophysis—Mesodinium* match–mismatch, Reloncaví Fjord, Chilean Patagonia

## Abstract

Species of the *Dinophysis acuminata* complex are the main cause of diarrhetic shellfish poisoning worldwide. These mixotrophs perform photosynthesis with plastids stolen from specific ciliate prey. Current transport models forecast advection of established populations, but modelling bloom development and maintenance also needs to consider the prey (*Mesodinium* spp.) of *Dinophysis*. Predator and prey have distinct niches, and *Dinophysis* bloom success relies on matching prey populations in time and place. During autumn 2019, red tides of *Mesodinium rubrum* in Reloncaví Fjord, Chile, were not followed by *Dinophysis* growth*.* The dynamics of *Mesodinium–Dinophysis* encounters during this and additional multiscale cases elsewhere are examined. Analogies with some classic predator—prey models (match–mismatch hypothesis; Lasker’s stable ocean hypothesis) are explored. Preceding dense populations of *Mesodinium* do not guarantee *Dinophysis* blooms if spatial co-occurrence is not accompanied by water column structure, which leads to thin layer formation, as in Lasker’s stable ocean hypothesis or if the predator growth season is over. Tracking the frequency of vacuolate *Dinophysis* cells, irrefutable signal of prey acquisition, with advanced *in situ* fluid-imaging instruments, is envisaged as a next-generation tool to predict rising *Dinophysis* populations.

## INTRODUCTION

Species of *Dinophysis* produce one or two kinds of lipophilic toxins: okadaic acid (OA) derivatives and pectenotoxins (PTXs). Toxins of the OA group cause diarrhetic shellfish poisoning (DSP), a gastrointestinal syndrome affecting consumers of shellfish contaminated with *Dinophysis* toxins (Yasumoto *et al*., 1980; Van Egmond *et al*., 1993). Detection of DSP toxins in shellfish above regulatory limits enforced by health authorities (DSP events) is a frequent cause of shellfish harvesting bans worldwide (Reguera *et al*., 2014) and the main threat to the Atlantic (Fernandes-Salvador *et al*., 2021) and Mediterranean (Accoroni *et al*., 2024) shellfish industry in Europe. *Dinophysis* species are a main target in shellfish monitoring programs, and improving the prediction of *Dinophysis* blooms is a priority in the most affected areas (Hariri *et al*., 2022; Ruiz-Villareal *et al*., 2022; Silva *et al*., 2023; Rosales *et al*., 2024).

Since the identification of *Dinophysis fortii* in 1980 as the causative agent of DSP intoxications, multiple attempts to establish *Dinophysis* species in culture failed (Maestrini, 1998). Twenty years later, in the era of molecular biology, Gustafson *et al*. (2000) demonstrated that the cryptophyte-like organelles in the phototrophic ciliate *M. rubrum* were sequestered from live prey belonging to the cryptophyte *Teleaulax acuta*. Finally, Park *et al*. (2006) established the first successful laboratory culture of *Dinophysis* (*acuminata* complex) fed on *M. rubrum*, itself fed *Teleaulax amphioxeia*. This kleptoplastidic three-step food chain was further used to establish cultures of other *Dinophysis* species: *D. acuta*, *D. caudata*, *D. fortii*, *D. infundibulus*, *D. ovum, D. tripos* (revised in Reguera *et al*., 2014) and, more recently, of *D. sacculus* (Riobó *et al*., 2013) and *D. norvegica* (Nagai *et al*., 2023). Park’s breakthrough paved the way for new research issues with *Dinophysis* as a model obligate mixotroph to address questions about the relative contributions of photosynthesis and heterotrophic nutrition (Hansen and Tillmann, 2020), the transformations of the kleptoplastids taken from *M. rubrum* prey (Kim *et al*., 2012b) and the extent and nature of their control by the host’s nuclear-encoded genes (Rusterholz *et al*., 2017; Hongo *et al*., 2019; Gaonkar and Campbell, 2023). *Dinophysis* species are nowadays classified as plastidic specialist non-constitutive mixotrophs (pSNCMs), i.e. they combine acquired phototrophy using plastids stolen from their prey, with phagotrophy of specific ciliate prey of the genus *Mesodinium* (Mitra *et al*., 2016). In turn, red (plastid-bearing) *Mesodinium* ciliates are pSNCMs that combine acquired phototrophy with phagotrophy of genus-specific (*Teleaulax/Geminigera*) cryptophyte prey.

Different growth phases of a microalgal population are promoted by distinct environmental factors. Thus, a model covering the full growth season of a microalgal species’ population should have several compartments corresponding to bloom development (including different motile and resting life cycle stages) and maintenance, transport and decline (Geohab, 2010). In the framework of early warning and forecasting of *Dinophysis* events, considerable progress has been achieved over the last decade with transport models (Velo-Suárez *et al*., 2010; Bedington *et al*., 2022; Hariri *et al*., 2022). These transport models have been useful in forecasting shoreward advection and offshore dispersal of already established populations during spin-down and spin-up phases of upwelling and along-shore transport at the end of the upwelling season (Ruiz-Villarreal *et al*., 2016; Monteiro *et al*., 2024; Rosales *et al*., 2024). However, these models do not incorporate biotic factors promoting intrinsic growth.

Given the nutritional adaptations of *Dinophysis* spp., realistic modelling of their bloom development and maintenance needs to consider their ciliate prey (*Mesodinium* spp.), the latter’s cryptophyte prey (*Teleaulax/Geminigera* clade) and their predator–prey interactions. Quantitative microvideography observations using a high-speed microscale imaging system (Jiang *et al*., 2018) confirmed that these interactions include: (i) chemoreception of *Mesodinium* prey, which, in turn, is alerted to the predator by mechanoreception and jump to escape (Hansen *et al*., 2013); (ii) prey are captured during arrest between consecutive jumps with an ejectile trichocyst-like peduncle and/or by mucus secretion (Mafra Jr. *et al*., 2016; Ojamäe *et al*., 2016); and (iii) prey are ingested by myzocytosis, a phagocytosis mode in which the predator sucks the content of the prey cell after piercing it with a feeding peduncle (Park *et al*., 2006). Most of the year, *Dinophysis* may be around the detection level (10^2^ cells · L^−1^) of conventional sampling methods used in monitoring. A major challenge is to understand how such low biomass populations of selective holoplankton succeed in catching their fast-swimming red *Mesodinium* prey. Further complexities emerge from the fact that the predator (*Dinophysis* spp.) and prey (*Mesodinium*) have different niches, i.e. they differ in the set of biotic and abiotic factors creating their optimal environmental conditions for survival and growth. For example, *M. rubrum* and its *Teleaulax* prey take up nitrates efficiently (new production) (Fiorendino *et al*., 2020; Gaillard *et al*., 2020), whereas members of the *D. acuminata* complex*,* high-affinity strategists adapted to grow in low nutrient environments, appear to prefer reduced (ammonia) and organic (e.g. urea) nitrogen (regenerated production) (Seeyave *et al*., 2009) and seem not to use nitrate (Hattenrath-Lehmann *et al*., 2015; García-Portela *et al*., 2020). Physiological experiments have pointed to availability of their ciliate prey as the crucial biotic factor that triggers the exponential growth of *Dinophysis* (Kim *et al*., 2008; Riisgaard and Hansen, 2009). Thus, *Dinophysis* bloom success relies on matching specific prey populations in time and place, i.e. when encounter rates are augmented by increased densities of co-occurring predator and prey populations. *M. rubrum* is a well-known red tide organism that exhibits striking red patches at the surface in summer as part of its daily vertical migration behaviour (Hansen and Fenchel, 2006; Moëller and Johnson, 2023). In contrast, *D. acuminata* populations reach densities above 10^4^ cells L^−1^ when cells aggregate, usually around vertical density gradients, forming a thin layer; dense populations are also found due to wind-driven advection (Díaz *et al*., 2013; 2019a). These advected populations, which often represent the annual maximum, are typically found during upwelling relaxation or in buoyant river plumes in late phases of the population growth. A match favourable to the predator will depend on the overlapping of predator and prey populations within a narrow window of space and time (González-Gil *et al*., 2010; Velo-Suárez *et al*., 2014).

There is only a recent attempt to address the dynamics of the *Teleaulax–Mesodinium–Dinophysis* food chain with a Nutrient-Phytoplankton-Zooplankton (NPZ) system dynamics model (Anschütz *et al*., 2022; Mitra, 2024). This model includes cyanobacteria (*Synechococcus*) as a potential food supply for *Mesodinium* prey, the constitutive mixotroph cryptophyte *Teleaulax,* and considers complexity required in the tunning of mixotrophic food chain models (Anschütz and Flynn, 2020).

The “critical time and space” constraints of *Mesodinium*–*Dinophysis* encounters within a narrow window of opportunity recall the pioneering observations on fish larval feeding, which paved the way to the classic match–mismatch hypothesis (MMH; Cushing, 1969; Cushing, 1990) and Reuben Lasker’s stable ocean hypothesis (SOH; Lasker, 1975; Lasker, 1978) of larval fish population dynamics.

Johan Hjort (1914), considered the father of modern fisheries science, was the first to suggest a connection between a successful fish year class and the planktonic food availability during the “critical period,” i.e. the time (days) from egg hatching to the first days of larval feeding, i.e. until the disappearance of the larvae yolk sac. Hjort’s hypothesis inspired other critical period-based hypotheses. David Cushing, in his MMH (Cushing, 1969), extended Hjort’s narrow critical period to the full larval development stage and stated that the reproductive success of a determined fish species depended on the extent to which the phenology of the prey (e.g. the phytoplankton production cycle) coincided with the timing of highest demand of the predator (e.g. spawning season). Lasker’s SOH (Lasker, 1975, 1978) focused on depth-resolved differences in larval feeding and its relation to plankton/chlorophyll vertical structure, which was, in turn, affected by the level of wind-driven mixing. Lasker described the feeding of anchovy larvae on *Gymnodinium splendens* (= *Akashiwo sanguinea*)*.* Effective larval feeding occurred when *Akashiwo* was aggregated in a high-density layer; larvae would starve if the layer was dispersed by wind mixing. Lasker events, the eponym created to honour Reuben Lasker, was coined to designate a time period of four calm days with wind strength below 6 m s^−1^, i.e. the time for the formation and maintenance of a high-density layer of *Akashiwo* for the “critical period” of time between egg hatching and yolk sac disappearance in anchovy larvae (Pauly, 1987).

**Fig. 1:**
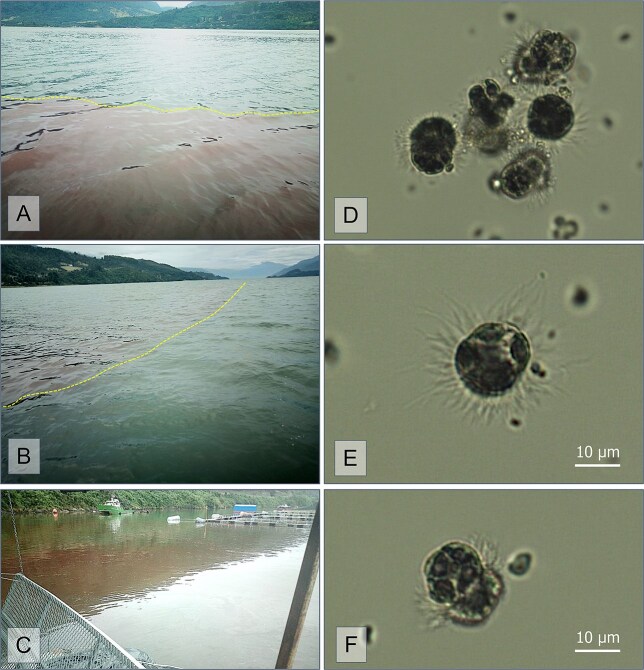
(**A**, **B**) *M. rubrum* red tide (the line delimits the boundary between the ciliate patches and the surrounding clear waters) observed near the head of RF, site of intense salmon farming (**C**) on 26 April 2019. (**D**, **E, F**) Light microscopy micrographs of Lugol's fixed specimen of *M. rubrum*. (Micrographs courtesy of C. Fernández-Pena)

In early autumn (April) 2019, brown patches of *Mesodinium* (up to 4.6 × 10^6^ cells · L^−1^) were observed in the innermost part of Reloncaví Fjord, Chile (Fig. 1). The ciliate bloom was not followed by the development of a *Dinophysis* population. This mismatch between *M. rubrum* and *D. acuminata* prompted the analysis of the depth-resolved distribution of environmental conditions during the autumn bloom of *Mesodinium*. Additional scenarios and time scales of observations of *Dinophysis*-*Mesodinium* matches/mismatches are examined here. These are (i) high-temporal-resolution multiannual tracking of dinoflagellate and ciliate in the Gulf of Mexico and multiannual monitoring in a Chilean fjord and a Portuguese coastal lagoon; (ii) spatial–temporal distribution in the Galician Rías during the full growth season and during a 2-week cruise; and (iii) intensive 24 h sampling surveys in Chilean Patagonia, the Baltic Sea and the Galician Rías. The results of this work highlight the time and space constraints affecting *Dinophysis* and *Mesodinium* blooms and indicate the suitability of monitoring *Mesodinium* populations, with traditional (cell counts) and advanced methods, as a key parameter for early warning and improved modelling of *D. acuminata* populations.

## MATERIAL AND METHODS

### Study area

**Fig. 2:**
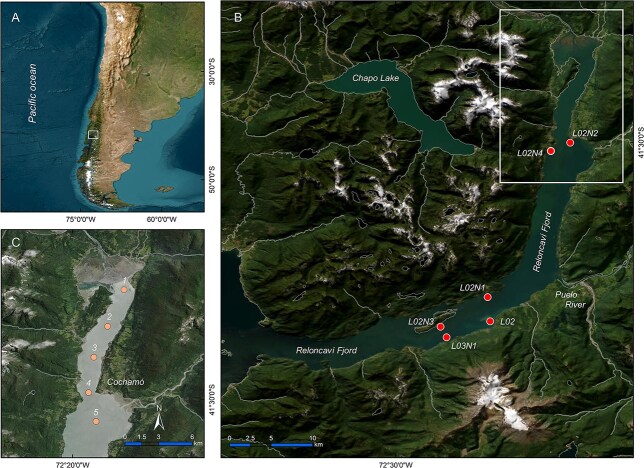
Maps showing: (**A**) South Cone of South America and location of the study area (the squared frame delimits RF area); (**B**) RF and IFOP monitoring sampling stations (the squared frame delimits the head of RF); and (**C**) the head of RF and sampling stations on a 12 km transect cruised on 26 April 2019.

The Reloncaví Fjord (RF), on the northern limit of the Chilean Patagonia, southern Chile (41 to 55° S), is one of the most extensive fjord and channel systems in the world (Fig. 2). A strong haline stratification is a permanent feature in this fjord due to heavy rainfall (~2700 mm y^−1^, 5000 mm in exceptional years) and riverine inflow from Puelo River (mean annual discharge ~650 m^3^ s^−1^ and peaks >5000 m^3^ s^−1^), which flows into the middle part of the fjord. Additional inputs come from the Petrohué (280 m^3^ s^−1^) and Cochamó (20 m^3^ s^−1^) rivers and glacier melting (Pickard, 1971; Castillo *et al*., 2016). Stratification patterns are extremely variable, in particular in the innermost areas subject to tidal energy perturbations (Valle-Levinson, 2010). These physical barriers have direct effects on the distribution of planktonic populations, (Geohab, 2010), including those of Harmful Algal Blooms (HAB) species (Alves De Souza *et al*., 2014; Díaz *et al*., 2021; Baldrich *et al*., 2022; Díaz *et al*., 2023). The complex bathymetry of this 55 km long fjord is characterized by a mean depth of 250 m and a maximal depth of 450 m close to the mouth (Fig. 2B). Wide tidal ranges (up to 7 m in spring tides) generate high surface flows into the fjord (Valle-Levinson *et al*., 2007; Aiken, 2008).

### IFOP phytoplankton monitoring

Monthly reports of phytoplankton cell counts from the RF from 2013 to 2023 were obtained from the IFOP (Fisheries Development Institute) Harmful Algal Bloom Management and Monitoring Program. In this program, integrated water-column samples (0–10 and 10–20 m) for analyses of phytoplankton are collected with a dividable hose sampler (Lindahl *et al*., 2007) at six sampling stations inside the fjord (Fig. 2B) and immediately fixed with Lugol’s solution (Lovegrove, 1960). For quantitative analyses of phytoplankton, 10 mL aliquots of the hose samples are left to sediment overnight and analysed under an inverted microscope (Olympus CKX41) using the method described in Utermöhl (1958). To enumerate large but less abundant species, such as *Dinophysis* spp., the whole surface of the chamber is scanned at a magnification of ×100, so that the detection limit is 100 cells L^−1^.

### Seasonal sampling and oceanographic cruise overview

#### Seasonal sampling

Measurements of physical and biological properties were carried out from January to July 2019 to study small-scale biophysical interactions promoting the development and aggregation of the main lipophilic toxin producers in RF, a hot spot of lipophilic toxin outbreaks. A fixed sampling point was established at the head of the fjord (Station 4; Fig. 2C). Sampling was done weekly between January and February, twice a month in March and once a month between April and July. Vertical profiles of temperature and salinity were obtained with an RBR Ltd., Otawa, Canada (RBR) Concerto 3 Conductivity Temperature Depth (CTD) profiler (http://www.rbr-global.com). The CTD probe was cast from surface to bottom (100 m depth) with a sampling rate of 8 Hz (eight measurements per second). CTD data were processed using the software provided by the manufacturer and depicted using Ocean Data View software version 5.1 (Schlitzer, 2015).

Water samples for microplankton counts and genetic analyses were collected with 5 L Niskin bottles every 2 m, from surface to 20 m. Aliquots were immediately fixed with neutral Lugol’s iodine solution (Lovegrove, 1960), to a final concentration of 0.5% and stored in the dark at room temperature. For nutrient analysis, bottle samples were collected every 4 m intervals from the surface to 20 m (six fixed depths). Dissolved inorganic nutrients [NO_3_^−^, NO_2_^−^, PO_4_^3−^ and Si (OH)_4_] were analysed from 500 mL seawater samples, stored at −20°C in High Density Polyethylene (HDPE) bottles, using a Seal AA3 AutoAnalyzer according to the methodology described in Grasshoff *et al*. (1983) and standard methods for seawater analysis (Kattner and Becker, 1991). Ammonia analyses were omitted for logistic reasons, i.e. the impossibility of ensuring analyses of this labile molecule very soon after collection in remote areas in Southern Chile.

Quantitative phytoplankton analyses (focused on *Dinophysis* and *Mesodinium*) were carried out according to the Uthermöhl method (Utermöhl, 1958), as described in the section IFOP phytoplankton monitoring.

#### Cruise transect

In early autumn (26 April) 2019, a 1-day cruise was carried out, starting at the head of RF. Sampling included five stations aligned in a 12 km transect (Fig. 2C). Vertical profiles of temperature and salinity to 50 m depth were measured, and water samples for micro-phytoplankton analyses were collected, fixed and stored following identical protocols as those used for the seasonal sampling and described in the previous section.

### Molecular characterization

Fixed cells of *M. rubrum* from samples collected at the head of RF on 26 April 2019 were imaged, individually picked with a microcapillary pipette, rinsed three times in droplets of distilled water and poured into 200 μL Polymerase Chain Reaction (PCR) tubes for immediate PCR amplification. Their partial plastid 23S rRNA gene sequence was amplified using universal primers p23Sr_f1 (5′-GGA CAG AAA GAC CCT ATG AA-3′) and 23Sr_r1 (5’-TCA GCC TGT TAT CCC TAG AG-3′) following Sherwood and Presting (2007). The amplification reaction mixtures (20 μL) were performed using Horse-Power™ Taq DNA Polymerase MasterMix (2×) (Canvax, Spain). DNA was amplified in an Eppendorf Mastercycler EP5345 (Eppendorf AG, New York, USA). An 8 μL aliquot of each PCR reaction was checked with agarose gel electrophoresis (1% tris acetate EDTA, TAE, 50 V) and GelRed™ nucleic acid gel staining (Biotium, Hayward, CA, USA). PCR products were purified with ExoSAP-IT™ (USB Corporation, Cleveland, OH, USA). Purified DNA was sequenced using the Big Dye Terminator v3.1 Reaction Cycle Sequencing kit (Applied Biosystems, Foster City, CA, USA) and migrated in an AB 3130 sequencer (Applied Biosystems) at the Centro de Apoyo Científico Tecnolóxico á Investigación (CACTI, Universidade de Vigo, Spain) sequencing facilities. The 23S rDNA sequence obtained in this study was deposited in GenBank (Acc.No. PQ136461). Comparison of target sequences against the GenBank database held by National Center for Biotechnology Information (NCBI), and further alignment and elaboration of phylogenetic trees using the maximum likelihood (ML) method and Bayesian estimation of phylogeny, were performed using MEGA genetics analysis software, version X (Kumar *et al*., 2018) and Geneious Prime (2023.0.4), respectively.

## RESULTS

### Oceanographic conditions

**Fig. 3:**
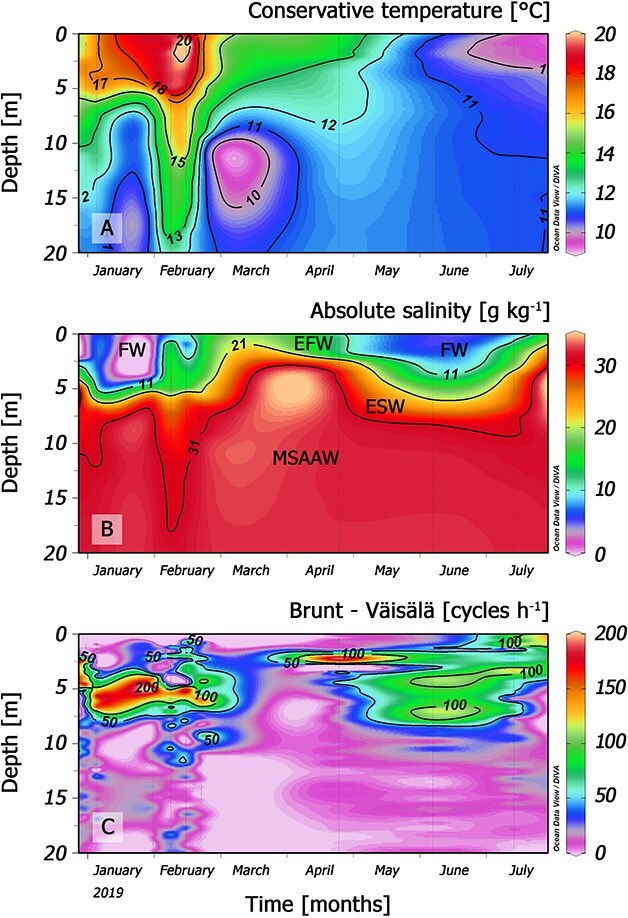
Vertical distribution of (**A**) conservative temperature (°C), (**B**) absolute salinity (g kg^−1^) and (**C**) Brunt–Väisälä frequency (cycles h^−1^) from 0 to 20 m at the fixed sampling station (St. 4) from January to July 2019.

During summer (January–February) a marked thermal stratification developed (Fig. 3), with a sea surface temperature (SST) maximum close to 20°C by mid-February (Fig. 3A). A two-layered water column structure was observed. An outflowing warmer and fresher estuarine water layer above and an inflowing colder and saltier layer of modified subantarctic water below 10–15 m, were separated by a pycnocline extending from 5 to 7 m depth (Fig. 3B). Cooling of SST led to an almost homogenous vertical structure in June and a thermal inversion (SST = 10°C) in July (Fig. 3A). Marked salinity gradients (~28 g kg^−1^/10 m) were a permanent feature throughout the study period (Fig. 3B). Density gradients and buoyancy frequency were maximal in the top 5 m (Brunt–Väisälä frequency, 200 cycles h^−1^) during summer associated with a brackish water layer at the surface (Fig. 3C).

### Inorganic nutrients

**Fig. 4:**
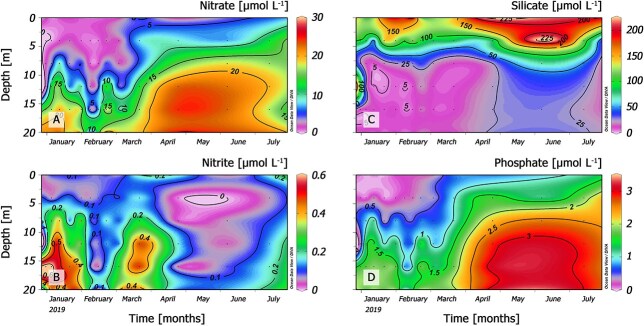
Vertical distribution of (**A**) nitrate (**B**) nitrite, (**C**) silicate and (**D**) phosphate concentrations (μM) from 0 to 20 m at a fixed sampling station (St. 4) from January to July 2019.

Concentrations of dissolved inorganic nutrients (nitrates, nitrites and phosphates) were extremely low in the surface layer (Fig. 4A, B, and D), but silicate was very high, exceeding 200 μmol L^−1^ in April 2019 (Fig. 4C). Maximum values of nitrates (25.3 μmol L^−1^; Fig. 4A), nitrites (0.64 μmol L^−1^; Fig. 4B) and phosphates (3.41 μmol L^−1^; Fig. 4D) were detected below 15 m with the exception of silicates, with a maximum of 255 μmol L^−1^ at the surface layer (Fig. 4C). An injection of bottom nutrient-rich water—mainly nitrate and phosphate— was observed from mid-March reaching maximum concentrations in May 2019.

### Patterns of pluriannual and seasonal distribution of *D. acuminata*—*Mesodinium* in RF

**Fig. 5:**
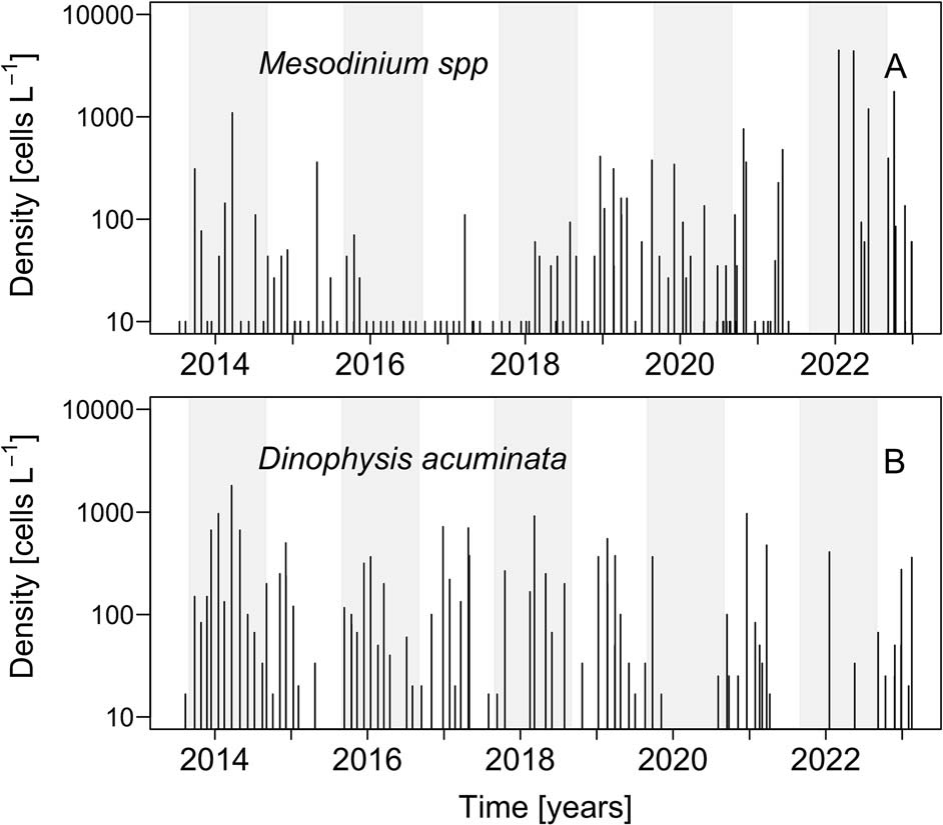
Time series (2013–2023) of monthly average (six stations, 0–10 m hose samples) of *Mesodinium* spp. and *D. acuminata* log_10_ cell densities in RF from 2013 to 2023.

From 2013 to 2023, time series of *Mesodinium* spp. and *D. acuminata* average cell densities from the six stations monitored by the IFOP showed different match–mismatch situations (Fig. 5). In 2014, both predator and prey peaks coincided in time and *Dinophysis* numbers (1.8 × 10^3^ cells L^−1^) exceeded those of *Mesodinium* (1.1 × 10^3^ cells L^−1^). From 2015 to 2017 (both years included), there was an apparent total mismatch: low densities of *Mesodinium* (100–350 cells L^−1^) were detected but still moderate (<10^3^ cells L^−1^) populations of *D. acuminata* occurred. In 2019, the year of this study, moderate populations of *Mesodinium* (300 cells L^−1^) and *Dinophysis* (500 cells L^−1^) coincided in mid-summer, but the year maximum of *Mesodinium* in mid-autumn occurred when the *Dinophysis* population had waned. Finally, in 2022, a moderate population of *Dinophysis* (400 cells L^−1^) coincided with a *Mesodinium* peak (4.4 × 10^3^ cells L^−1^) in January.

**Fig. 6:**
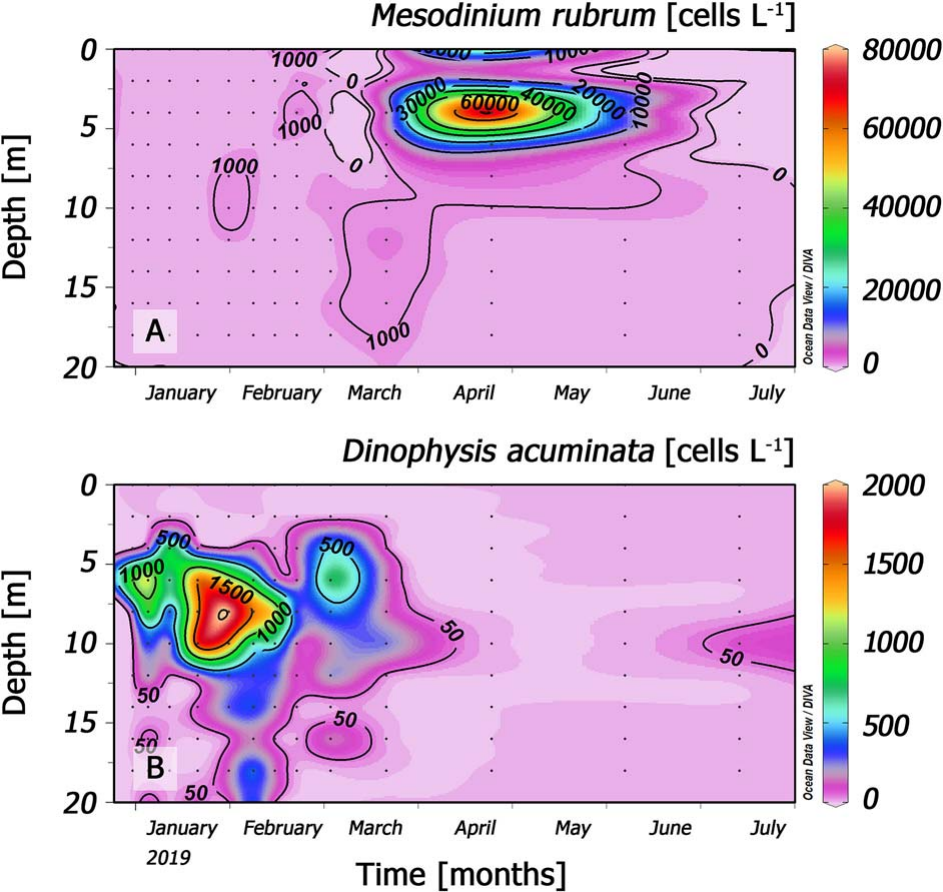
Vertical distribution of (**A**) *M. rubrum* and (**B**) *D. acuminata* from 0 to 20 m at a fixed sampling station (St. 4) from January to July 2019.

Seasonal variability was studied with a higher-vertical-resolution bottle sampling at specific depths in the fixed station (St. 4) (Fig. 6). In summer (January–March), moderate populations of *Mesodinium* (<10^3^ cells L^−1^) and the *Dinophysis* annual maximum coincided. *D. acuminata* cells were detected within temperature (~11–13°C) and salinities (~11–32) ranges common for the season. The cell maximum at the depth of maximal density gradient was limited by the boundary between the Estuarine Freshwater layer (EFW, 11–21) and the Estuarine Salty Water layer (ESW, 21–31). A more intense peak of *Mesodinium* was detected in early autumn (26 April), with 85 × 10^3^ cells L^−1^ at 4 m depth when *Dinophysis* was no longer detected. Cell densities declined after that peak but were above detection levels until the end of the study in July. Higher numbers were always found at the surface, but there were always cells detected below 10 m. *D. acuminata* cells were never detected in the surface freshwater layer (FW < 11) from 0.5 to 2 m, originated from the inflow of the Puelo and Petrohue rivers throughout the year.

### Distribution of *Mesodinium* in a cruise transect with *Dinophysis* under detection levels

**Fig. 7:**
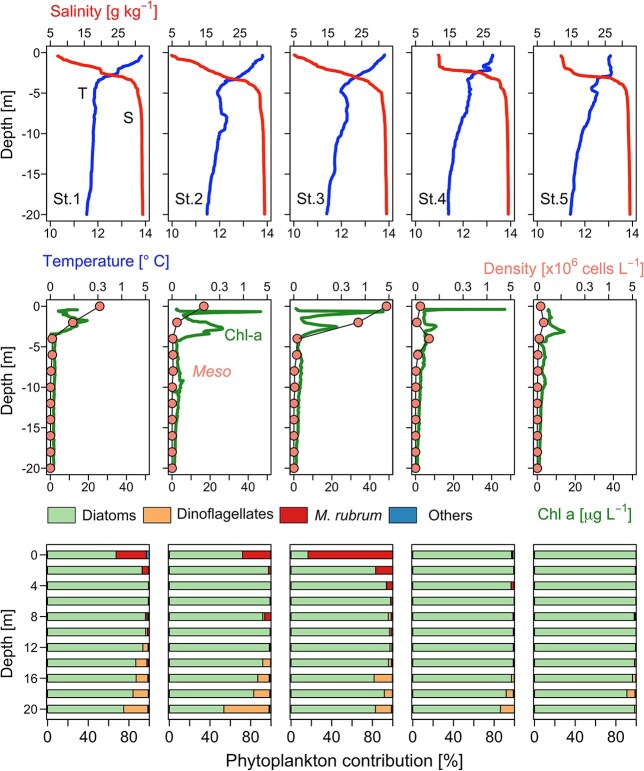
Vertical distribution (0–20 m) of: top panel, CTD profiles of temperature and salinity; mid-panel: chlorophyll *a* fluorescence and *M. rubrum* cell density (cells L^−1^); bottom panel: contribution (%) of different phytoplankton groups to the microphytoplankton assemblages in the five stations of the 12 km transect sampled on 26 April 2019.

On 26 April 2019, vertical profiles of temperature, salinity, fluorescence and *Mesodinium* numbers at each of the five stations from the transect indicated that the *Mesodinium* red tide extended horizontally from the head of the fjord to the physical barrier created by the strong haline front formed at the mouth of the Puelo River (Fig. 7). CTD and phytoplankton vertical profiles showed that at station 3, the *Mesodinium* red tide coincided with the chlorophyll maximum. *Mesodinium* cells were aggregated in a top (0–2 m) warmer (14°C) and very brackish (salinity < 5) water layer. A salinity gradient of ~10 m^−1^ was recorded in this layer (5 at 0 m, 25 at 2.5 m). In all stations sampled, *Dinophysis* cells were under detection levels.

### Plastid diversity of *M. rubrum*

**Fig. 8:**
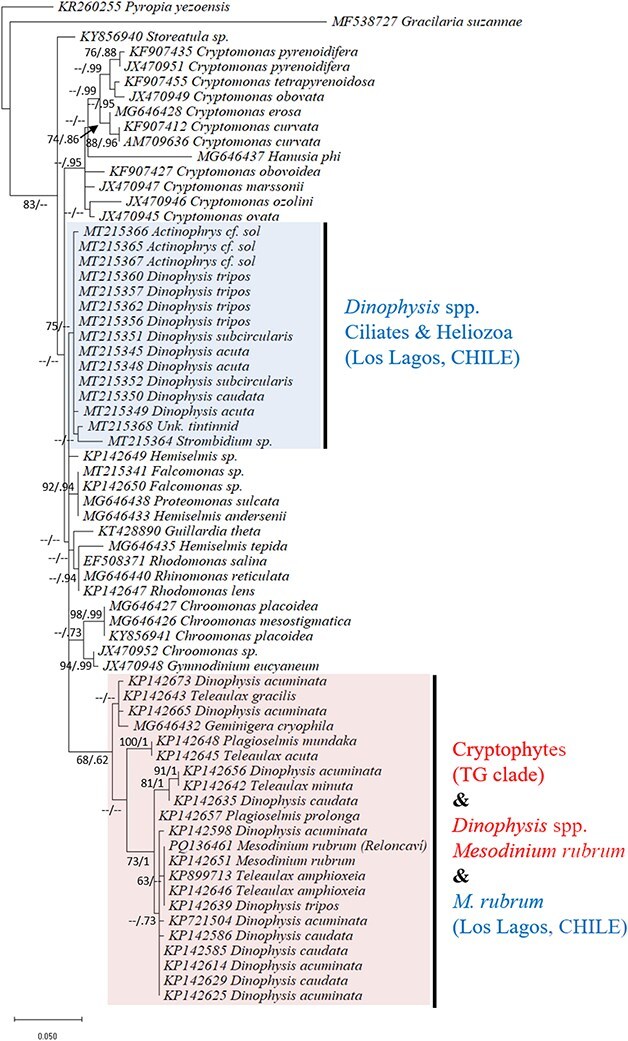
Plastidial 23S rDNA phylogeny (ML method) of single cells of *Mesodinium* cf. *rubrum* isolated at the head of Reloncaví Fjord on April 26, 2019. Support levels of internal nodes are bootstrap (ML, estimated from 1000 replicates) and posterior probability values (Bayesian analyses). Hyphens indicate low statistical support <60/.60, respectively.

Partial plastid 23S rDNA sequences were retrieved from ciliate *Mesodinium* cf. *rubrum* specimens individually picked from samples collected during the intense bloom in April 2019 (Fig. 1). Their phylogenetic analyses placed the sequences from *M. rubrum* within cryptophytes belonging to clade IV (*Teleaulax/Geminigera*) (Fig. 8). This group included *Teleaulax, Geminigera*, *M. rubrum* (isolated from RF) and *Dinophysis* spp. and *M. rubrum* sequences from the Iberian Peninsula.

## DISCUSSION

In the new classification of planktonic protists (Mitra *et al*., 2016), red planktonic *Mesodinium* ciliates and dinoflagellates of the genus *Dinophysis* have been both classified as plastidic specialist, non-constitutive mixotrophs (Mitra *et al*., 2016). *Dinophysis* is a highly selective (specialist) predator of red *Mesodinium* species (*M. rubrum/M. major* complex); *Mesodinium* is also a genus-selective predator of crytophycean (*Teleaulax/Geminigera* T/G clade) flagellates (Myung *et al*., 2011; Peltomaa and Johnson, 2017; Hansen and Tillmann, 2020). The success of *Dinophysis* bloom development relies to a large extent on its prey availability. Studies on phytoplankton succession with a focus on harmful species have shown the distinct phenology of *Mesodinium* spp. and *D. acuminata,* the latter increasing in later stages of the phytoplankton succession when regenerated production prevails (Álvarez-Salgado *et al*., 2011; Velasco-Senovilla *et al*., 2023; Ladds *et al*., 2024). Physiological experiments with *D. acuminata* and *M. rubrum* cultures have described their different adaptations to light intensity, nitrogen sources and windows of temperature and salinity for growth and survival (Hansen *et al*., 2013; Hansen and Tillmann, 2020).

Laboratory studies with both species have shown that they tolerate wide ranges of temperature, salinity and irradiation. Nevertheless, their optimal environmental windows for growth are very narrow (Fiorendino *et al*., 2020; Rial *et al*., 2023).

### *Mesodinium* and *D. acuminata* mismatches can be related to their different physiological traits

*Mesodinium* not only steals the plastids of its prey but also their nuclei (Johnson and Stoecker, 2005; Hansen and Fenchel, 2006; Johnson *et al*., 2007) and efficiently takes up dissolved nitrate and phosphate (Hansen *et al*., 2013). Field sampling in the Baltic Sea linked decreased phosphate concentrations in the surface layer to a preceding bloom of *Mesodinium* in spring, highlighting its efficient nutrient consumption (Lips and Lips, 2017). *Mesodinium* can also survive without feeding for long periods of time (up to 3 months) at low irradiance (Johnson and Stoecker, 2005; Smith and Hansen, 2007) and, according to Stoecker *et al*. (2009), alternates “bloom forming” with “slow-growth-maintenance” life styles.

In the case of *Dinophysis*, only the plastids are taken from the prey, but several nuclear encoded genes enable *Dinophysis* to control them for a few divisions (Rusterholz *et al*., 2017). In cultures, *D. acuminata* seems unable to take up nitrates (Hattenrath-Lehmann and Gobler, 2015; García-Portela *et al*., 2020). Following incubations of bloom populations with radiolabelled nitrogenous compounds, this species was classified as a “high-affinity strategist,” adapted to grow with low levels of ammonium and urea (regenerated production) (Seeyave *et al*., 2009). Niche-based analyses in an upwelling system (NW Spain) revealed that *D. acuminata* is very tolerant, i.e. able to survive under a wide range of environmental conditions, but also very “marginal,” e.g. grows optimally only within a very narrow range of conditions (Velasco-Senovilla *et al*., 2023). These observations agree with those from the Gulf of Mexico reported by Harred and Campbell (2014).

Physiological experiments with *Dinophysis* cultures have shown that specific prey (*M. rubrum*) availability and their good nutritional status and abundance (predator:prey ratio) are the key biotic factors to trigger the intrinsic growth (division) rate of *D. acuminata* (Kim *et al*., 2008; Riisgaard and Hansen, 2009) and the food chain *Dinophysis–M. rubrum–T. amphioxeia,* the only successful approach tested (Nagai *et al*., 2020).

These observations have led monitoring programs in shellfish aquaculture regions seriously affected by *Dinophysis* to add *Mesodinium* spp. to their target list. Nevertheless, simple information on cell densities of predator and prey (quantity) is weak without additional information on their respective quality. Also, simplistic conclusions may be reached if the limitations of each sampling procedure are not considered. We discuss these two problems in the context of the present case study in addition to results available from other studies with a variety of spatio-temporal scales and environmental conditions. The leading question of the discussion is: does monitoring of the potential prey, *Mesodinium*, help to predict *Dinophysis* blooms?

### Multiscale field distribution illustrating mismatch of *M. rubrum* and *D. acuminata* populations

#### Multiannual variability in a fjord, a subtropical estuarine bay, and a coastal lagoon subject to seasonal upwelling

*RF, Chilean Patagonia* (this study). In the 2013–2023 time series from RF, correlations between predator and prey populations were very poor. The exception was 2014, the year of *D. acuminata* maximum in the series when their annual cell maxima coincided. In this study, cell counts of *Dinophysis* and *Mesodinium* from six stations were averaged to dampen tidal effects in microscale distributions inside the fjords. Still, monthly sampling is clearly insufficient/inadequate to explore the dynamics of *Mesodinium* and *Dinophysis* encounters. Furthermore, hose samples from 0 to 10 m dilute the ciliate and dinoflagellate aggregates. Differences over one order of magnitude can be observed between hose- and bottle-sample estimates (Escalera *et al*., 2012). This problem is exacerbated in fjord systems where extreme density gradients and the cell maxima may be located in the top 5 m.

Alves De Souza *et al*. (2019) used a niche-based approach (realized subniche, Within Outlying Mean Index, WitOMI (Dolédec *et al*., 2000, Karasiewicz *et al*., 2020) to analyse the same time series of *Dinophysis* but from 2008 to 2017. These authors concluded that despite the strong effects of local climate variability, *D. acuminata* in RF was subject to strong biological constraints, probably as a result of the low cell densities of its prey (*Mesodinium* cf. *rubrum*) usually observed in the area. Blooms of *D. acuminata* occurred in 2008, 2011 and 2014, mostly during Austral southern summer months. An exception was in spring 2008 when a bloom of this species was observed in the head of the fjord, but there is no information on *Mesodinium* counts available from those days.

*Port Aransas, Texas, Gulf of Mexico*. The dataset with the best time resolution available for *Dinophysis* and *Mesodinium* is that from the Gulf of Mexico (Harred and Campbell, 2014) recorded *in situ* with an Imaging FlowCytobot (IFCB). The instrument, moored at 4 m depth in a shallow subtropical bay (Port Aransas, TX), monitored and quantified *Mesodinium* spp. and *D. ovum* (a member of the *D. acuminata* complex) from 2007 to 2012. In 4 out of 5 years, there were peaks of abundance of *Mesodinium* preceding *Dinophysis* with a time lag of 1–3 months. The exception was in winter 2008, when the two peaks overlapped. By February 2008, a cell record of *D. ovum* (2 × 10^5^ cells L^−1^) was recorded. This led to the first report of a harmful *Dinophysis* bloom in the Gulf of Mexico (and in the USA) (Campell *et al*., 2010) and the first shellfish harvesting closure caused by contamination of the oyster beds with diarrhetic shellfish poisoning (okadaic acid) toxins (Deeds *et al*., 2010).

Correlation coefficients between predator and prey abundance in the time series were very low (*r* < 0.5), and there was a case (in 2011–2012) when a peak of *Dinophysis* was not preceded by any substantial increase of *Mesodinium* above background levels. This last event and observations of a wide range in the size of *Mesodinium* cells led the authors to suggest that species other than *M. rubrum* might be present to serve as alternative prey. Another important remark was that in most cases, *Mesodinium* and *Dinophysis* bloom initiation or rising of their cell densities occurred during or just after high tide, supporting the view of an offshore origin of their inoculum populations. Both *Mesodinium* and *Dinophysis* were detected the whole year round in Port Aransas, but different environmental windows were identified for their bloom initiation: *Mesodinium* (T: 23–29°C, S: 30–34) from January to the end of May had a longer season than *Dinophysis* (T: 11–19°C, S: 28–33), from February to April.

*In situ* monitoring with the IFCB provides a unique time resolution, but the main limitation of this study was that all the information came from a single depth (4 m) at a fixed station.

*Aveiro Lagoon, Portugal*. Moita *et al*. (2016) analysed 10 years of weekly samples from Ría de Aveiro (Northern Portugal), a shallow coastal lagoon connected to shelf waters. Samples were collected at a shallow station (5 m) with Niskin bottles. Sampling time was always 1 h before high tide so as to monitor shelf waters that entered the ria. *Mesodinium* and *D. acuminata* maxima co-occurred although *Mesodinium* usually persisted when the *D. acuminata* growth season was over (Moita *et al*., 2016). The authors suggested that different-sized *Mesodinium* cells observed through the season possibly corresponded to the co-occurrence of more than one species of *Mesodinium* prey: *M. rubrum* and the other considerably larger red *Mesodinium* species that probably corresponded to *M. major,* recently erected by García-Cuetos *et al*. (2012). The longer growth season of *Mesodinium* spp. in Aveiro, in agreement with the observations in Port Aransas and in RF, would explain cases of *Mesodinium* peaks observed when *Dinophysis* was no longer detected. Consistent match–mismatch situations in Aveiro, in which tidal effects were considered (Moita *et al*., 2016), lead to the suspicion of false positive/negative in other case studies when samples are taken with no consideration of the tidal situation (low tide, intense spring tides) in coastal embayments with high flushing rates and tidal range.

**Fig. 9:**
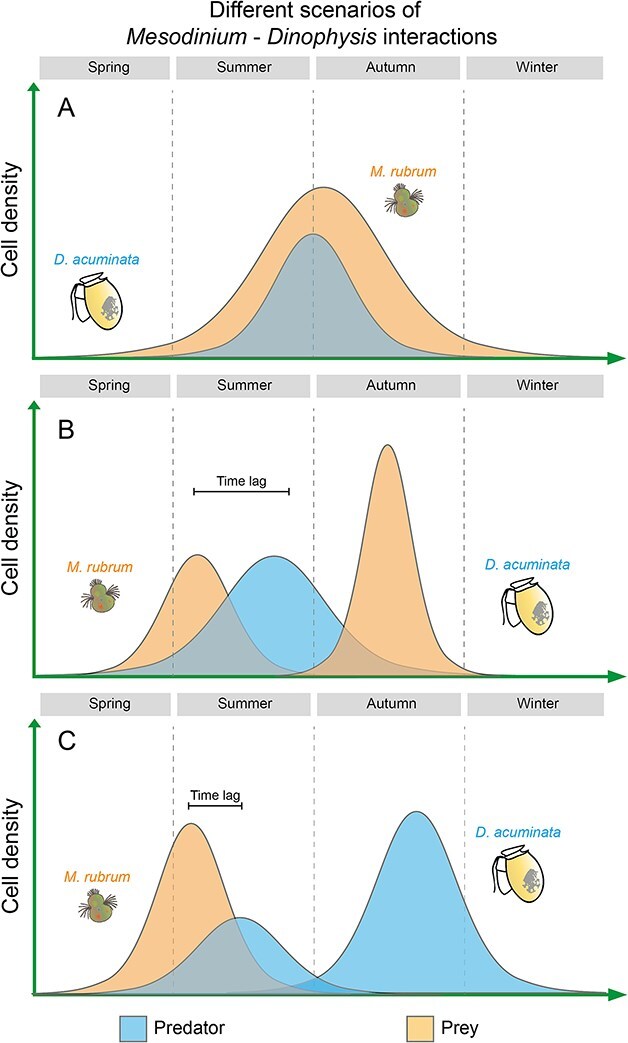
Different scenarios of *Mesodinium*–*Dinophysis* encounters: (**A**) full match of *Mesodinium* and *Dinophysis* populations maxima. (**B**) Spring–summer time-lagged maxima of *Mesodinium* and *Dinophysis* and a second un-matched autumn peak of *Mesodinium.* (**C**) Spring–summer time-lagged maxima of *Mesodinium* and *Dinophysis* and a second un-matched autumn peak of *Dinophysis.*

Despite differences in sampling devices and resolution in the three case studies above, they coincided with pointing to the full match of the ciliate and dinoflagellate populations in years of exceptional blooms of *Dinophysis* (Fig. 9A) and to unexplained *Dinophysis* peaks in the absence of prey (advected populations or sampling problems?) (Fig. 9B).

#### Seasonal variability of Mesodinium and Dinophysis encounters in a fjord and in a coastal upwelling ria

*Chilean Patagonia Fjords*. In RF (this study), moderate populations of *Mesodinium* and *Dinophysis* coincided in summer 2019. A bloom of *Mesodinium* spp. was recorded in the autumn when *D. acuminata* growth season was over. *Mesodinium* cells were detected from 0 to 20 m, but most cells were aggregated in the top 2.5 m of a very brackish (S: <5) water layer formed from the input of Puelo and Petrohué rivers in a year of heavy positive anomalies in precipitation and river outflow. Earlier studies in the area described the niche of *D. acuminata* within the EFW layer (salinity 17–20) and optimal temperature ~15°C; the end of thermal inversion in the water column (colder in/at the surface) preceded the initiation of the growth season (Baldrich *et al*., 2021, 2023). In the present case study of an autumn bloom of *Mesodinium*, high cell densities of *Mesodinium* were observed in the top layer (0–6 m) where nutrients were depleted, except high silicate concentrations from the rivers (Fig. 5C). This may indicate that at the time of sampling, *Mesodinium,* an efficient consumer of inorganic nitrogen (nitrates) and adapted to grow with higher light intensities than *Dinophysis*, had already depleted the inorganic nutrients available in the system. This pattern has been observed after the spring bloom of *Mesodinium* in the central Gulf of Finland (Lips and Lips, 2017) and in the northern Galician Rías (Álvarez-Salgado *et al*., 2011) where *D. sacculus* blooms at a later stage of the succession when there is a predominance of regenerated production. In contrast, in the present study, nitrate and phosphate were depleted in the top 8 m in summer, when *Dinophysis* year/annual maximum (1.8 × 10^3^ cell L^−1^) developed in the layer of maximal density gradient (around *S* = 21, boundary between the EFW and the ESW) and *Mesodinium* exhibited a second maximum in the autumn, when nutrient levels and temperature raised again. A possible explanation here is that the extreme low salinity values prevented the occurrence of *Dinophysis;* released predation pressure on the euryhaline *Mesodinium* prey enhanced the magnitude of the predator/prey mismatch (Fig. 9C).

*The Galician Rías, NW Spain*. The seasonal co-occurrence of *Mesodinium* spp. and *D. acuminata* populations from spring to autumn was described in 2007 from weekly sampling at a hotspot of DSP in the Galician Rías (Velo-Suárez *et al*., 2014). These authors monitored not only the number of *Dinophysis* cells but also their nutritional status. Well-fed *Dinophysis* were recognized from their vacuolation (just fed) and their content of starch granules. Key features in the bloom initiation were stratification and upwelling–relaxation dynamics together with the presence, for the first time in the season, of high numbers of *M*. cf *rubrum*. Peaks of *M. rubrum* and *D. acuminata* did not always have the same vertical or horizontal distributions, but their moving patches eventually met. Observations of an abrupt increase in *D. acuminata* cell numbers with a high proportion of vacuolated cells during downwelling suggest that *Mesodinium*–*Dinophysis* encounters and good feeding on *Mesodinium* prey occurred offshore, probably in the upwelling front in shelf waters (González-Gil *et al*., 2010).

Velo-Suárez *et al*. (2014) noticed that peaks in the frequency of vacuolated cells were only recorded on a few occasions, suggesting that *D. acuminata* is prey-limited most of the time but does not require a constant supply of prey for long-term survival.

#### Circadian resolution in Mesodinium and Dinophysis distribution

High-frequency depth-resolved measurements of co-occurring populations of *Mesodinium* spp. and *D. acuminata* have revealed some features of their circadian rhythms. Observations from 36- to 48-h surveys in the Galician Rías, Western Iberia (Díaz *et al*., 2019b), Puyuhuapi fjord, Chilean Patagonia (Baldrich *et al*., 2021) and the Baltic Sea (Sjöqvist and Lindholm, 2011), have shown the co-occurrence of *D. acuminata* and *Mesodinium* spp. cell maxima in time and place but differing in swimming behaviour. In the three cases, *D. acuminata* was aggregated in a thin layer and showed no signs of daily vertical migration, but *Mesodinium* swam downwards at night and upwards during the day. Thus, the ciliate and dinoflagellate maxima matched in the early morning and noon in the Baltic Sea (Sjöqvist and Lindholm, 2011), at noon in the Galician Rías (Díaz *et al*., 2019b) and at night in the Chilean fjords (Baldrich *et al*., 2021). This common behaviour of *Dinophysis* in stratified systems suggests that predator and prey overlap when *D. acuminata*, using the angler’s strategy, intercepts *Mesodinium* spp. in their migrations. *Dinophysis* aggregation in thin layers seems to create optimal conditions for predator–prey encounters requiring a minimum distance for prey-efficient detection and capture (Jiang *et al*., 2018). A different behaviour was reported by Villarino *et al*. (1995) during extremely calm weather and water column stability in the Galician Rías in August. On that occasion, *D. acuminata* migrated from the surface during the first hours of light, to ~10 m depth in the evening and night period, coinciding with the vertical migration of *M. rubrum*. Finally, in a study comparing results from 24-h surveys in Puyuhuapi and Pitipalena fjords, the presence and distribution of *Mesodinium* spp. was not related to that of *D. acuminata*, and it was evident that prey was not a limiting factor (Baldrich *et al*., 2023).

### Match–mismatch of *Mesodinium*–*Dinophysis* populations and their hypothetical causes

Biological factors, such as the quality and quantity of prey, and abiotic factors, e.g. environmental conditions with contrasting effects on predator and prey phenology, have been proposed to interpret cases of *Mesodinium* and *Dinophysis* mismatch.

#### Alternative prey hypothesis

Observations of dense populations of *Dinophysis* in the absence of *M. rubrum* or a wide range in the size of *Mesodinium* cells throughout the *Dinophysis* growth season (Harred and Campbell, 2014; Moita *et al*., 2016) and cases of *Dinophysis* isolates with plastids corresponding to cryptophycean clades other than the TPF clade (Stern *et al*., 2014; Díaz *et al*., 2020) or even to other microalgal groups (Rial *et al*., 2015; Nishitani and Yamaguchi, 2018) has led different authors to question the species-specific selection of *M. rubrum* as the only possible live prey for *D. acuminata*. Potential plastid-bearing ciliates other than *Mesodinium* have been suggested as alternative prey (Stoecker *et al*., 2009; Reguera *et al*., 2012). To resolve these uncertainties, site-specific genetic characterization (partial sequencing of plastidic and ribosomal DNA) of the suspected prey and the predator (*Dinophysis* spp.) needs to be compared. However, studies of systematic sequencing of plastidic 23S DNA in field populations of *Dinophysis* spp. and co-occurring hypothetical ciliate prey throughout their growing season are rare (Reguera *et al*., 2024).

Johnson *et al*. (2017) found that *M. rubrum* and *M. major*, the red *Mesodinium* species described by García-Cuetos *et al*. (2012) were but different genetic variants conforming to the *M. major*/*M. rubrum* species complex. A recently erected species, *M. annulatum* (Nam *et al*., 2024), was found to correspond to subclade B, one of the eight subclades of the *M. rubrum/M. major* complex, five of which have not been assigned yet to a described species of *Mesodinium.* Mixotrophic cultures of *M. annulatum* were established with the *Mesodinium*–*Teleaulax* food chain used with *M. rubrum* from the same location. The same response may be expected as new species of the *M. rubrum/M. major* complex are described.

Most plastid sequences obtained from single-cell isolates of *Dinophysis*, mainly specimens from the *D. acuminata* complex (*D. acuminata, D. sacculus, D. ovum*) matched those found in *M. rubrum/M. major* complex and/or in their cryptophyte prey of the genera *Teleaulax*/*Geminigera* (TG clade). This clade corresponds to what was earlier called the TPG clade until the demonstration by Altenburger *et al*. (2020) that *Plagioselmis* (n) was but the haploid form of *Teleaulax* (2n). The co-occurrence of field populations of *M. rubrum* and *M. major* with the same plastid sequences has been confirmed in the Galician Rías (Rial *et al*., 2015) and isolates of *M. major,* which occurs later than *M. rubrum* in Danish waters, were established in culture with *T. amphioxeia* (Drumm *et al*., 2021).

In Patagonia, Southern Chile, *D. acuminata* strains with different toxin profiles have been reported (Díaz *et al*., 2022), but there is no information available on plastidic sequences of Chilean strains of this species. Recently Paredes-Mella *et al*. (2022) established the first cultures of *D. acuminata* from Chile grown with *M. rubrum*-fed *T. amphioxeia*. Ciliate and cryptophyte strains were isolates from Japanese coastal waters. The plastidic sequences of field isolates of *M. rubrum* from the RF (this study) coincided with that from the Galician specimens (Fig. 8). These findings led to the assumption that in the northern Patagonian fjords, *M. rubrum* is the main prey of *D. acuminata*, but *M. major* reported in the region (Johnson *et al*., 2017) or even other undescribed species of the complex will need to be tested. More sequencing is needed to discover whether different morphotypes of *Mesodinium* in a given place correspond to different growth phases of the same or different species. In any case, differences in size and plastidic content of the *Mesodinium* prey were associated with a proportional response in *Dinophysis* field populations (Harred and Campbell, 2014) and relevant differences in growth rate and final yield in mixotrophic cultures of *Dinophysis* (García-Portela *et al*., 2018; Smith *et al*., 2018). Eventually, a small proportion of *Dinophysis* specimens isolated from the same region have been found to contain plastids of multiple taxonomical origins (e.g. chlorophytes, florideophytes, raphidophytes and stramenopiles); these plastids could have been stolen from prey other than *M. rubrum/M. major* complex (Hackett *et al*., 2003; Kim *et al*., 2012a; Rial *et al*., 2015). Nevertheless, the occasional detection of plastid sequences corresponding to ciliate genera other than *Mesodinium* has to be interpreted with caution and doesn’t necessarily mean that they can sustain long-term growth of *Dinophysis* spp.

Recently, field specimens of *Dinophysis* (*D. acuta, D. caudata, D. tripos* and *D. subcircularis*) other than *D. acuminata* from Chilean Patagonia showed the same partial plastidic sequence as co-occurring protozoan *Stenophrys* cf. *sol* and ciliates of the genus *Strombidium* (Díaz *et al*., 2020). These observations added support to the alternative prey hypothesis, with *Strombidium* sp. as the putative prey in that particular case. Unlike the *M. rubrum* complex, species of the ciliate genus *Strombidium* have cryptophyte-like kleptoplastids corresponding to the *Rhodomonas/Rhinomonas/Storeatula* (RRS) clade (Hoef-Emden, 2008). There is only one previous report of RRS clade–like plastids found in picked cells of *D*. cf. *acuta* from Scotland (Stern *et al*., 2014), but *Strombidium* spp. were the predominant ciliates co-occurring with *D. norvegica* blooms in the Baltic Sea. Nevertheless, more plastid sequencing from *Dinophysis* isolates of species other than *D. acuminata* throughout the seasonal growth period is crucial to address this issue. In the meantime, exciting new results are coming from *Strombidium* species recently established in culture, such as *S*. cf. *basimorphum* (Maselli *et al*., 2021) and *S. rassoulzadegani* (Grzywacz *et al*., 2014), and the dynamics of the plastids stolen from their prey.

#### Environmental conditions and match–mismatch of Mesodinium–Dinophysis populations

From the species characteristics given above, it can be expected that *Mesodinium* growth starts in earlier phases of the annual phytoplankton succession when “new production” dominates. *Dinophysis* belongs to later stages of succession when nitrates and phosphates are exhausted and when dominance of high affinity-strategist mixotrophs is favoured. However, the two species may reach a seasonal maximum at the same time if located in different water layers. Under stratified conditions, they may form patches and/or thin layers that eventually match. Such a scenario might correspond to years of “bloom success” with a full match of the two populations. The mechanism of encounter, i.e. thin layers of *Dinophysis* intercepting migratory cells of *Mesodinium*, would be as described in the circadian encounter section. The same strategy applies to *Mesodinium* (preying on *Teleaulax*), which has been described as a “sit-and-wait predator”; it waits for nearby prey and then stuns and captures them with its feeding tentacles (Moëller and Johnson, 2023).

The key question is: can we assume that high-density patches of *Mesodinium* will trigger increased *D. acuminata* numbers? The answer will depend on the where, when, and how of the *Dinophysis* population status. In other words, only if there exists a suitable size population of *Dinophysis* in feeding mode and a water column structure that allows for adequate predator–prey encounter rates. This reasoning is valid when what matters is the prediction of the intrinsic population growth rate at the beginning of the growth season. The case studies discussed here illustrate dense spring–summer blooms of *Dinophysis* when there was a full match of predator and prey. There were also cases of late blooms of *Mesodinium* when *Dinophysis* growth was over. So how do we interpret sudden peaks of *Dinophysis* with no preceding detection of prey?

Another possibility would be that the peak corresponded to wind-driven populations in their final transport phase. These stationary-phased populations, often prey-starved, do not occur following the early warning of a preceding peak of *Mesodinium.* Still, they might be at the peak of their toxin content per cell and their maximal potential to contaminate shellfish (Pizarro *et al*., 2013). These wind-driven advected populations are commonplace in coastal areas subject to seasonal upwelling regimes where they cause the most damaging toxic HAB events (Sordo *et al*., 2001; Trainer *et al*., 2010).

#### Dinophysis–Mesodinium encounters in light of some classical predator–prey models

A real challenge is to unveil the mechanisms controlling the functioning of *Dinophysis* unique mixotrophic food chain and its variability. In other words, the conditions where the intersection of the predator and prey populations promote their encounters. Here, new approaches are sought in light of some classic predator–prey models, used in fish population dynamics, which include density-dependent (aggregation) behaviour and time and space constraints.

In the MMH (Cushing, 1969), emphasis is put on *time*; the degree of match or mismatch in the timing of larval production (time of spawning season) and the production of their food (seasonal phytoplankton cycle) was proposed to explain interannual changes in year-class strength in fish populations. The time of fish spawning is rather constant and controlled by an internal clock of the adult fish, but the initiation of phytoplankton production is controlled by the onset of thermal stability and increased light penetration (“critical depth hypothesis”; Sverdrup, 1953 and others) and is more variable. Data from the Continuous Plankton Recorder have shown that in the Northeast Atlantic and North Sea the date of the spring phytoplankton peak may vary up to 6 weeks (Colebrook, 1982).

As in the MMH, maximal densities of *Dinophysis* blooms are attained when there is a good match of predator (*Dinophysis*) and prey (*Mesodinium*) cell maxima. However, there are important differences between the two cases. In the MMH, the margin of match/mismatch time to contribute to the success/failure of larval development is measured in days. In contrast, the initiation of the *Dinophysis* and *Mesodinium* growth season is linked to and co-varies with the phytoplankton production cycle (Álvarez-Salgado *et al*., 2011; Velo-Suárez *et al*., 2014; Ladds *et al*., 2024). Starvation is not such a rigid temporal constraint for mixotrophic *Dinophysis* and *Mesodinium* with adaptations to switch from phototrophic to phagotrophic modes of nutrition (opportunistic behaviour) or to persist as fugitive, stress-tolerant species in the system (Velasco-Senovilla *et al*., 2023).

In Ruben Lasker’s “SOH,” Lasker’s (1975) emphasis is placed on *space* (fine-scale vertical structure of phytoplankton). Extensive larval feeding occurs when the phytoplankton food is aggregated forming a subsurface chlorophyll maximum layer. Strong winds associated with storm events mix the water column, disaggregate layers of high prey concentration and reduce the survival of first-feeding larvae. “Lasker events,” originally defined as 4 days of calm sea (wind velocity below 10 m s^−1^), are the time windows that allow anchovy larvae to feed effectively on aggregated *Akashiwo sanguinea*. Further testing of the hypothesis with other fish species has shown that the duration of the “critical period” of first larval feeding, ranging from 4 to 10 days, is fish species–specific, so the duration of Lasker events needed for prey aggregation (cell densities to be defined) varies between production systems (Turley and Rykaczewski, 2019). In other words, the vertical structure promoting “thin layers of phytoplankton” (TLP) with suitable composition and persistence to secure early larval feeding of each fish species needs to be defined.

Lasker’s SOH and the related key parameters to test it [metrics of “wind events” (storms) or calm periods; rates of larval mortality; and description of the vertical structure of plankton in the water column] are similar to those needed to unveil the mechanisms behind TLP formation and of *Dinophysis–Mesodinium* encounters. Both need layers of a high density of cells aggregated at a certain density gradient depth. However, in the SOH, the prey (dinoflagellates) is the fixed part that needs to be aggregated for efficient predator (fish larvae) feeding, whereas, in the *Dinophysis–Mesodinium* case, the fixed part is the predator (*Dinophysis*), which needs to be aggregated to intercept more efficiently its fast-swimming prey (*Mesodinium*). The mortality rate of the prey (*Mesodinium)* would be the most difficult parameter to measure, but increasing rates of vacuolate cells of *Dinophysis* may be a good proxy of the predator feeding rate.

### Management and prediction implications and the potential of *in situ* imaging flow cytometry for *Dinophysis* monitoring

Data on *Mesodinium*–*Dinophysis* show that confirmed *Mesodinium* prey numbers may prove a good tool to predict *Dinophysis* bloom initiation provided a *Dinophysis* inoculum is present. Also, rapid increases in *Dinophysis* cell numbers may be predicted with models that consider not only predator and prey numbers but also the adequate vertical structure that will secure prey detection and capture. Here, we suggest that an unbiased observation to predict imminent intrinsic growth (increased division rates) in *Dinophysis* populations is the number that has actually eaten the prey and not the abundance of potential prey. The detection of vacuolate cells is irrefutable proof of effective feeding. However, quantification under the light microscope of vacuolated cell frequencies in a fixed sample is very time-consuming and has only been carried out in a few studies (Velo-Suárez *et al*., 2014).

### Biological interactions are the dark side of the moon in HAB ecology, in particular in low biomass toxic HABs of mixotrophs in which we do not the nature of their prey

*In situ* imaging flow cytometry is a promising tool increasingly used to alleviate the workload associated with phytoplankton identification and quantification by conventional microscopy methods (Dashkova *et al*., 2017). These field-deployable instruments acquire images of suspended particles (seston) and discriminate planktonic cells on the basis of fluorescence and optical cell scatter measurements (Thyssen *et al*., 2008).

The first report of a harmful *Dinophysis* bloom was the result of a serendipitous observation with an IFCB aiming to detect *Karenia* blooms in the Gulf of Mexico (Campell *et al*., 2010). Exciting new information has been obtained *in situ* about the behaviour and biotic interactions of *Dinophysis* in the last few years. These include observations on the phased mating of *Dinophysis* gamete pairs as well as on *Dinophysis* grazers (Ladds and Gobler, 2024; Sung-Clarke *et al*., 2023). Recently, Houliez *et al*. (2023) explored new possibilities to track the physiological status of *Dinophysis* individuals. *Dinophysis* and *Mesodinium* are non-constitutive mixotrophs, and prey ingestion, or the lack of it, affects their size and shape, as well as their pigment quota and their “darkness” (Jacobson and Andersen, 1994). Starved *Dinophysis* are more difficult to detect on the basis of their fluorescence, but differences in “darkness” might be easier to detect in the cells’ light scatter. Houliez *et al*. (2023), recommended alternating light-scattering and fluorescence-triggering methods to monitor non-constitutive mixotrophs with an IFCB. This idea was pursued by Ladds *et al*. (2024) who developed a flowcytobot application to calibrate the “darkness” of the mixotroph cells and quantify them. This kind of application opens new possibilities to automate observations on the nutritional status of *Dinophysis* and other mixotrophic microalgae in field populations.

## CONCLUSIONS

*Dinophysis acuminata* and its prey *Mesodinium* are both holoplanktonic, prey-selective mixotrophs, which perform photosynthesis with plastids stolen from their respective prey organisms. Both show a high tolerance to prey starvation but each has distinct physiological and behavioural traits which explain why *Mesodinium* growth season is longer, and a peak of *Mesodinium* does not guarantee a bloom of *D. acuminata*.

In the few document case studies available, years with high numbers of *D. acuminata* occurred when: i) at the seasonal scale, the population growth curve maxima of *Dinophysis* and its *Mesodinium* prey coincided (full match); ii) at the microscale, when water column structure favoured aggregation of *Dinophysis* populations (no vertical migration mode) and intercept vertically migrating patches of *Mesodinium*, leading to a high proportion of well-fed (vacuolate) *Dinophysis* cells.

High numbers of *D. acuminata* can result from cell aggregation resulting from in situ growth, or from physically advected populations. The latter is frequent in the final transport phase of the population when *Dinophysis* is no longer feeding. Modelling *Dinophysis* population growth requires inclusion of predator-prey interactions of the two mixotrophic protists. These interactions are far more complex than those of simple unidirectional phototroph-heterotroph food chains. The classic Lasker “Stable Ocean Hypothesis”, in which the critical requirement is water column structure, may be a suitable approach to parameterize a useful *Dinophysis*-*Mesodinium* “upside down” predator-prey relation, in which a swimming prey is grazed by a “predator-in-waiting”. Vacuolate cells are the irrefutable signal of effective *Dinophysis* feeding which triggers intrinsic growth. Tracking the frequency of vacuolate cells, although a cumbersome task with conventional microscopy methods, may prove to be the most suitable parameter to measure, in addition to in situ division rates. Recent results with advanced in situ fluid-imaging instruments, using cell “fullness” (side scattering) as a proxy for vacuolation are promising. They are envisaged as a next-generation tool to predict rising *Dinophysis* populations.

## Data Availability

The data underlying this article are available in the article and also from the corresponding author, B.R., upon request.
